# Transcriptome analysis of potential candidate genes and molecular pathways in colitis-associated colorectal cancer of *Mkp-1*-deficient mice

**DOI:** 10.1186/s12885-021-08200-0

**Published:** 2021-05-25

**Authors:** Ahmed Hammad, Zhao-Hong Zheng, Akhileshwar Namani, Mohamed Elshaer, Xiu Jun Wang, Xiuwen Tang

**Affiliations:** 1 Department of Biochemistry and Department of Thoracic Surgery of The First Affiliated Hospital, Zhejiang University School of Medicine, Hangzhou, 310003 People’s Republic of China; 2 Department of Pharmacology, Zhejiang University School of Medicine, Zhejiang University, Hangzhou, 310058 People’s Republic of China; 3 Present address: Department of Biotechnology, Institute of Science, GITAM, Visakhapatnam, 530045 India

**Keywords:** Mkp-1, Colitis, Colorectal cancer, RNA sequencing, Biomarker

## Abstract

**Background:**

The nuclear phosphatase mitogen-activate protein kinase phosphatase-1 (MKP-1) is a key negative regulator of the innate immune response through the regulation of the biosynthesis of proinflammatory cytokines. In colorectal cancer (CRC), which is induced mainly by chronic inflammation, Mkp-1 overexpression was found in addition to disturbances in Mkp-1 functions, which may play a role in cancer development in different types of tumors. However, the potential molecular mechanisms by which Mkp-1 influences CRC development is not clear. Here, we performed global gene expression profiling of Mkp-1 KO mice using RNA sequencing (RNA-seq) to explore the role of Mkp-1 in CRC progression using transcriptome analysis.

**Methods:**

Azoxymethane/dextran sodium sulfate (AOM/DSS) mouse models were used to examine the most dramatic molecular and signaling changes that occur during different phases of CRC development in wild-type mice and *Mkp-1* KO mice. Comprehensive bioinformatics analyses were used to elucidate the molecular processes regulated by Mkp-1. Differentially expressed genes (DEGs) were identified and functionally analyzed by Gene Ontology (GO), Kyoto Enrichment of Genes and Genomes (KEGG). Then, protein-protein interaction (PPI) network analysis was conducted using the STRING database and Cytoscape software.

**Results:**

Persistent DEGs were different in adenoma and carcinoma stage (238 & 251, respectively) and in WT and *MKp-1* KO mice (221& 196, respectively). *Mkp-1* KO modulated key molecular processes typically activated in cancer, in particular, cell adhesion, ion transport, extracellular matrix organization, response to drug, response to hypoxia, and response to toxic substance. It was obvious that these pathways are closely associated with cancer development and metastasis. From the PPI network analyses, nine hub genes associated with CRC were identified.

**Conclusion:**

These findings suggest that MKp-1 and its hub genes may play a critical role in cancer development, prognosis, and determining treatment outcomes. We provide clues to build a potential link between Mkp-1 and colitis-associated tumorigenesis and identify areas requiring further investigation.

**Graphical abstract:**

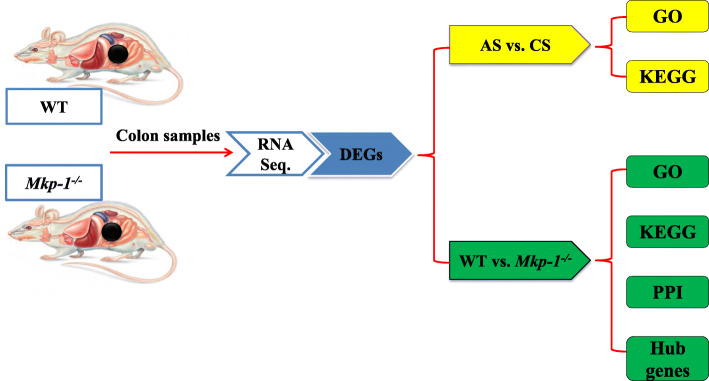

**Supplementary Information:**

The online version contains supplementary material available at 10.1186/s12885-021-08200-0.

## Introduction

Colorectal cancer (CRC) is the third most common malignancies worldwide [1]**,** with more than 1.2 million new cases diagnosed annually [2]. According to Cancer statistics in China, 2015, there were approximately 376,000 new patients with CRC and 191,000 new CRC death, accounting for a fifth of malignant tumor incidence and mortality [3].

CRC metastasis is a common phenomenon and the major lethal cause of CRC, as approximately 50% of patients with CRC die within 5 years due to extensive metastasis [4]. CRC developed through a multistep process from normal epithelium to adenoma and adenocarcinoma, which can eventually metastasize to different organs [5]; it was shown that the transition of CRC cells from early stages to late stages (metastasis) is associated with a significant change in the cellular gene expression profile [6]. These genetic alterations have led to the emergence of the novel strategies for personalized medicine programs [7, 8]. Moreover, the correlation between colorectal inflammation and increased colitis-associated tumorigenesis has been identified in some epidemiological and clinical studies [9, 10], which is characterized by high mortality and is closely associated with inflammatory bowel disease [11].

Mitogen-activated protein kinase phosphatases (MAPK phosphatases, MKPs) are a family of proteins that have been identified as major negative regulators of MAPK activities, which act as potential regulators of cell growth and survival in physiological and pathological processes [12]. Specifically, Mkp-1, also named dual-specific phosphatase 1, is a nuclear phosphatase and a key negative regulator in the innate immune response [13, 14], by deactivating p38 and c-Jun N-terminal kinases by dephosphorylating threonine and tyrosine, which play pivotal regulatory roles in the biosynthesis of proinflammatory cytokines. Notably, in colon cancer, Mkp-1 was overexpressed in the early phases of carcinogenesis [15, 16], and it impaired the response to cetuximab-based treatment in patients with metastatic colon cancer [17]. Furthermore, the therapeutic effect of camptothecin in colon cancer cells was improved through inhibition of Mkp-1 activity [18]. These studies suggest that Mkp-1 may be an attractive target for colon cancer therapy [15]. The involvement of Mkp-1 in cancer therapy resistance has also gained prominence, making Mkp-1 a potential target for anti-cancer therapy [12].

Indeed, the role of Mkps in cancer has been less well-defined despite increasing evidence that disturbances in Mkp-1 function may play a role in cancer development. According to recently published papers, there are controversial roles of Mkp-1 in cancer development and progression. Although some studies have shown that the gain of Mkps expression has been associated with drug resistance, cancer progression, and poor patient prognosis [19], others have shown that the loss of Mkps induces cancer development or progression due to the loss of negative regulation of MAPKs [12].

Conclusively, the main cause of CRC is the disturbance in immune responses and some metabolic pathways, in addition to the main roles of Mkp-1 involved in the regulation of immune responses. We aim to highlight the role of Mkp-1 in CRC in this study using transcriptome analyses associated with bioinformatics data mining tools, using RNA-seq, which provide an opportunity to simultaneously analyze a huge number of genes/targets, and explore the potential genes and molecular signaling pathways regulated by Mkp-1.

## Material and methods

### Chemicals

Unless otherwise stated, all chemicals purchased from Sigma-Aldrich Co., Ltd. (St. Louis, MO, USA). Antibodies were obtained from Santa Cruz Biotechnology (Dallas, TX, USA). DSS (36–50 kDa) was obtained from MP Biomedicals (Aurora, OH, USA).

### Animals and animal ethics

BALB/c background WT mice were purchased from Shanghai Laboratory Animal Center (Chinese Academy of Sciences, Shanghai, China). After obtaining the permission from Bristol-Myers Squibb Co. (New York, NY, USA), Professor Andrew R. Clark (University of Birmingham, UK) provided us *Mkp-1*^*−/−*^ mice on a C57BL/6 background [20]. BALB/c background *Mkp-1*^*−/−*^mice were produced by eight back-crossings of C57BL/6 *Mkp-1*^*−/−*^mice with BALB/c WT mice. Genotypes of *Mkp-1*^*−/−*^mice were routinely identified by RT-PCR and western immunoblotting. Mice were maintained in the cages (VM370B64S9 cages with size 370x157x180 mm, Suhangtech, Jiangsu, China) in groups of four under standard conditions of ventilation, temperatures (22 ± 30 C), and lighting (light/dark: 13 h/11 h) and kept under observation for 1 week prior to experimentation. Drinking water and standard pellets diet were provided ad libitum throughout the study. All animal experiments were performed with the approval of the Laboratory Animal Ethics Committee of Zhejiang University. Animal studies were conducted in compliance with the ARRIVE guidelines [21, 22]. All the studies were performed in accordance with relevant guidelines and regulations. A complete ARRIVE Guidelines checklist is included in Additional file 1.

### Induction of colitis-associated CRC

**Fig. 1:**
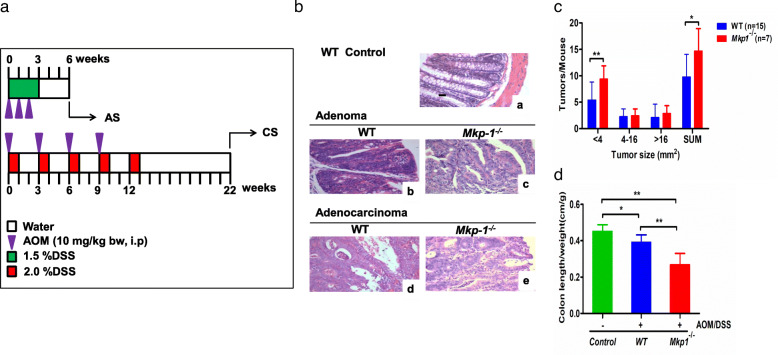
AOM/DSS induced colitis associated colon cancer. **(A)** Experimental protocols for induction of colitis-associated Adenoma and Adenocarcinoma in WT and *Mkp-1*^*−/−*^ mice. BALB/c WT and *Mkp-1*^*−/−*^ mice received AOM/DSS for 6 weeks to induce AS, and for 22 weeks to induce CS. **(B)** Representative images of H&E staining of colonic tumor sections in the WT (a), at the end of AOM/DSS treatment in AS (b, c), and CS (d,e). Normal colons from WT mice without any treatment considered as control (× 200). **(C)** Tumor size per mouse. SUM represented the sum of tumor sizes of all lengths. Values are mean ± SEM; *P < 0.05, **P < 0.01 vs WT mice. **(D)** Colon length (the length of tumor colon (cm) / the weight of mice (g) prior to AOM/DSS administration at the beginning of experiment) (n for control = 4, for WT =15, and for *Mkp-1*^*−/−*^ = 7), values are mean ± SEM; *P < 0.05, **P < 0.01 vs *c*ontrol mice. AS, adenoma stage; CS, adenocarcinoma stage; WT, wild type

The azoxymethane (AOM)-initiated and dextran sodium sulfate (DSS)-promoted mice model has been widely used to simulate the pathogenesis observed in patients with CRC [23]. In fact, carcinogenesis was induced by the injection of an AOM (inducing aberrant crypt foci caused by DNA damage) and DSS mixed in the drinking water (inducing colitis caused by inflammatory damage in the epithelial lining of the colon) in mice [24]. Therefore, two colitis-associated CRC models of adenoma and adenocarcinoma, which represent the initial and later stages of colon cancer, respectively, were induced using a AOM/DSS treatment strategy modified from that of Suzuki et al. [25] as we were previously described [26]. The protocol for the induction of adenoma stage (AS) is illustrated in Fig. 1. Briefly, 5–6-week-old male BALB/c mice were injected with AOM (10 mg/kg i.p.) once per week for the first 3 weeks. During this period, mice were exposed to drinking water containing 1.5% DSS and were left to drink normal water for 3 weeks. For the carcinoma stage (CS), 5–6-week-old-old male mice were also injected with AOM (10 mg/kg i.p.), exposed to drinking water containing 2% DSS for 1 week, and then left to drink normal water for 2 weeks. This treatment was repeated for three additional cycles. Finally, the mice were left to drink water containing 2% DSS for 1 week, followed by normal water for 9 weeks. At the end of each period of AS and CS, mice were sacrificed by cervical dislocation. The colon was dissected as described previously [13], and hematoxylin and eosin (H&E) staining was performed for histopathological analysis of tissue sections (3-μm) [27, 28].

### RNA-seq analysis

RNA-seq analysis and library were described previously [26]. Indeed, The present study involved two main categories including wild type that subdivided to four subgroups; wild type control (WT-C) for adenoma stage, wild type adenoma stage (WT-AS), wild type control (WT-C) for carcinoma stage, wild type carcinoma stage (WT-CS), which we used them in our previous work but in different purposes [26], and Mkp-1 KO type that subdivided to four subgroups; Mkp-1 KO control (*Mkp-1*^*−/−*^C) for adenoma stage, Mkp-1 KO adenoma stage (*Mkp-1*^*−/−*^AS), Mkp-1 KO control (*Mkp-1*^*−/−*^C) for carcinoma stage, Mkp-1 KO carcinoma stage (*Mkp-1*^*−/−*^ C S). At the end of the experiment, Animals (*n* = 3 for each group) were sacrificed and distal colonic epithelial tissue samples from each group were dissected and subjected to total RNA extraction using TRIzol reagent (Invitrogen, Carlsbad, CA) according to the manufacture’s instruction. RNA quality was assessed using an Agilent Bioanalyzer (Agilent Technologies, Palo Alto, CA). The RNA was sheared and reverse transcribed using random primers to obtain cDNA, which was used for library construction. Illuminam RNA-Seq libraries were subsequently performed using the SMARTer Stranded RNA-Seq Kit (Clontech, Mountain View, CA) according to the manufacturer’s instructions. All statistical analyses were carried out with HTSeq v0.6.1; after removing transcriptionally inactive genes (read count per million < 1) from raw RNA sequencing gene counts, we obtained high confidence gene counts. For gene expression analysis, the matched reads were calculated and then normalized to reads per kilobase of exon model per million mapped reads (FPKM) [29, 30]. The RNA-seq data of raw and processed files have been deposited in NCBI Gene Expression Omnibus, accession number GSE164960.

### Screening for differentially expressed genes (DEG)

The DEGs of WT-AS vs. WT-C, WT-CS vs. WT-C, *Mkp-1*^*−/−*^ AS vs. *Mkp-1*^*−/−*^ C, and *Mkp-1*^*−/−*^ CS vs. *Mkp-1*^*−/−*^ C were identified using the R statistical software package (www.r-project.org). The cutoff criteria were |logFC| ≥1.5 and adjusted *P*-value < 0.05. Moreover, overlapping DEGs between different groups have been identified by UpSet plots [31].

### Functional enrichment analysis

The enrichment analyses using GO terms (http://www.geneontology.org/) and KEGG pathways (http://www.genome.jp/kegg/) data for gene sets were performed using the NIH Database for Annotation, Visualization and Integrated Discovery (DAVID) web tool [32]. This tool can provide a functional interpretation of huge gene lists derived from genomic studies. A Benjamini *p*-value of< 0.05 was used in the analysis.

### Protein-protein interaction (PPI) network analysis

To further investigate the molecular mechanism of knockout in CRC, specific DEGs of *Mkp-1*^*−/−*^ groups were used to construct the PPI network by utilizing the biological online database tool (Search Tool for the Retrieval of Interacting Genes, STRING, http://stringdb.org) [33] to identify and explore the interactions among them. A combined score of > 0.4 (high confidence score) was considered significant, and the PPI network was visualized using Cytoscape software (Version3.5.1) [34]. The hub genes/proteins, a small number of crucial nodes for the protein interactions in the PPI network, were chosen with a centrality degree > 5.

### Cluster analysis for hub genes

The expression profile of hub genes was performed using heatmaper, a web-based tool [35].

### Survival analysis

Hub genes in various cohorts were subjected to a survival analysis using PROGgeneV2 [36] and PrognoScan [37].

### Statistical analyses

Statistical analyses were carried out using Stata7 for Windows (Stata Corp LLC, College Station, TX, USA). Student’s t-test was used to compare the two groups. Groups of more than two were compared using one-way ANOVA followed by Bartlett’s test. Spearman’s correlation was used to analyze the two ranked variables. One mouse was classified as one experimental unit. A *P*-value < 0.05 was considered statistically significant.

## Results

### *Mkp-1* deficiency promotes tumorigenesis in AOM/DSS-induced colitis-associated CRC

Treatment of *Mkp-1*-deficient mice with AOM/DSS (Fig. 1A) triggered the colitis, which was confirmed by a significant decrease in the length of colon in KO mice compared to WT one (Fig. 1D). Histopathological examination using H&E staining showed that AOM/DSS treatment for 6 weeks induced adenoma stage in WT and KO mice compared to normal WT colon, representing the early stage of tumorigenesis. Treatment for 22 weeks induced adenocarcinoma stage, representing the late stage of tumorigenesis (Fig. 1B). In addition, the number of smaller tumors (< 4 mm^3^) per mouse in KO (*P* value < 0.01) mice compared to WT mice increased; the numbers of medium-sized (4–16 mm^3^) and large (> 16 mm^3^) tumors per mouse were similar in the KO and WT mice (Fig. 1C).

### Novel molecular pathways identified in carcinoma stages

RNA-Seq provides an opportunity to simultaneously analyze a huge number of genes and distinguish between molecular pathways of early stage of CRC (adenoma) and late stage of CRC (adenocarcinoma), thus we analyzed the profiles of DEGs between diseased mice and control mice following the cutoff criteria fold-change |logFC| > 1.5 and *P*-value < 0.05 by ANOVA test. A library with size-normalized count for the specimen was generated by making volcano plots for the DEGs (Additional file 2: sFig. 1).

**Fig. 2:**
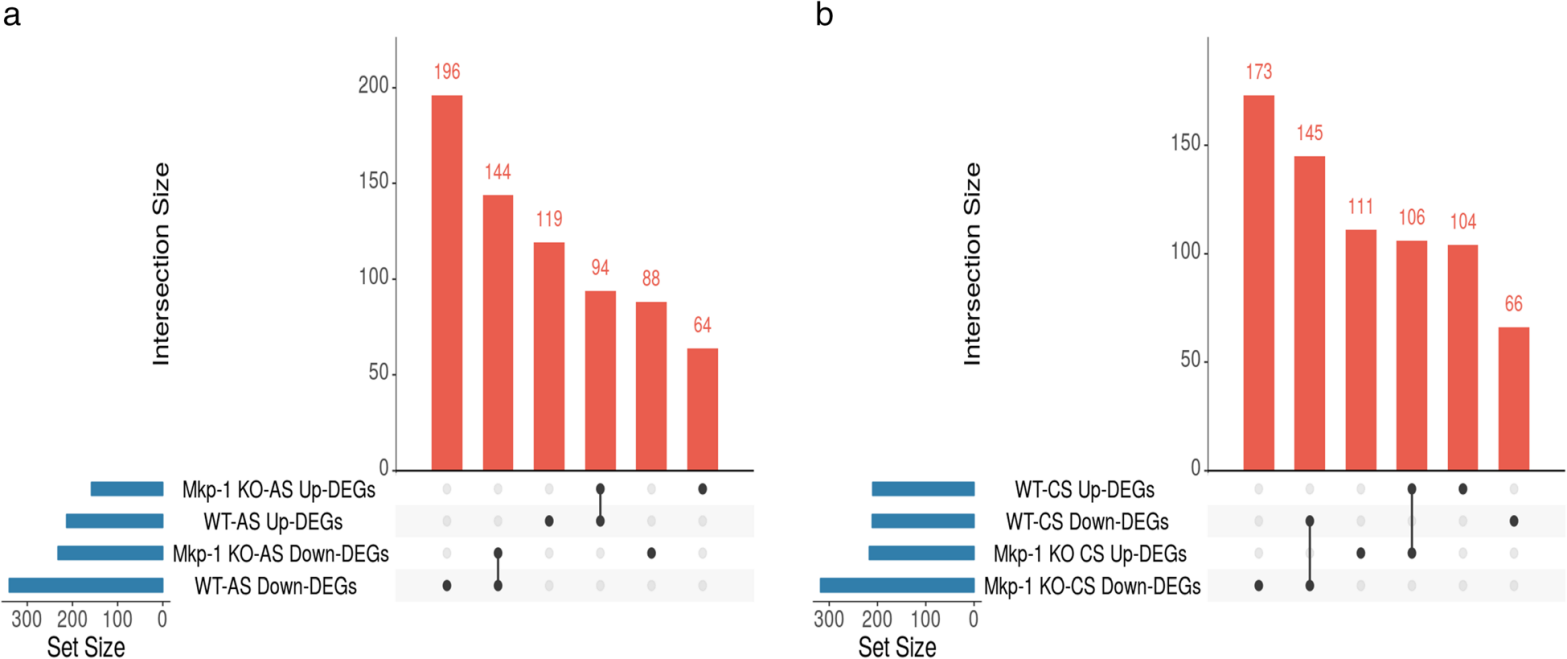
DEGs of early and late stages of tumors in the colon of mice administered with AOM/DSS. UpSet plots showing upregulated DEGs (Up-DEGs) and downregulated DEGs (Down-DEGs) overlapping in AS and CS for WT & KO mice. **a** Adenoma, AS **b** Carcinoma, CS. Each horizontal bar on the left represents the total number of DEGs identified in each set. Each vertical bar shows the number of genes that have been detected in each of the intersection sets, indicated by the connected points in the lower part of the plot

Data from RNA-seq were analyzed to obtain overlapping DEGs in adenoma and carcinoma stages. We identified 94 upregulated and 144 downregulated common DEGs in adenomas (553 DEGs in wild type divided into 213 upregulated and 340 downregulated and 390 DEGs in KO type divided to 158 upregulated & 232 downregulated) (Fig. 2a). In carcinomas, 106 upregulated and 145 downregulated DEGs overlapped (421 DEGs in wild type divided into 210 upregulated & 211 downregulated, and 535 DEGs in KO type divided into 217 upregulated & 318 downregulated) (Fig. 2b). Indeed, the numbers of persistent DEGs in carcinoma is slightly higher than in adenoma, showing that the progression of CRC affects the profile of expressed genes. (All the gene IDs and fold changes are listed in Additional files 3 and 4).

As we mentioned above, CRC metastasis is a common phenomenon and the major cause of death, and the transition of CRC cells from early stages to late stages (metastasis) is associated with a significant change in the cellular gene expression profile. Therefore, we compared signaling pathways regulated in adenomas and carcinomas to identify the novel pathways that are considered specific for carcinoma; and the findings can be used in improving the early diagnosis and treatment of CRC.

**Fig. 3:**
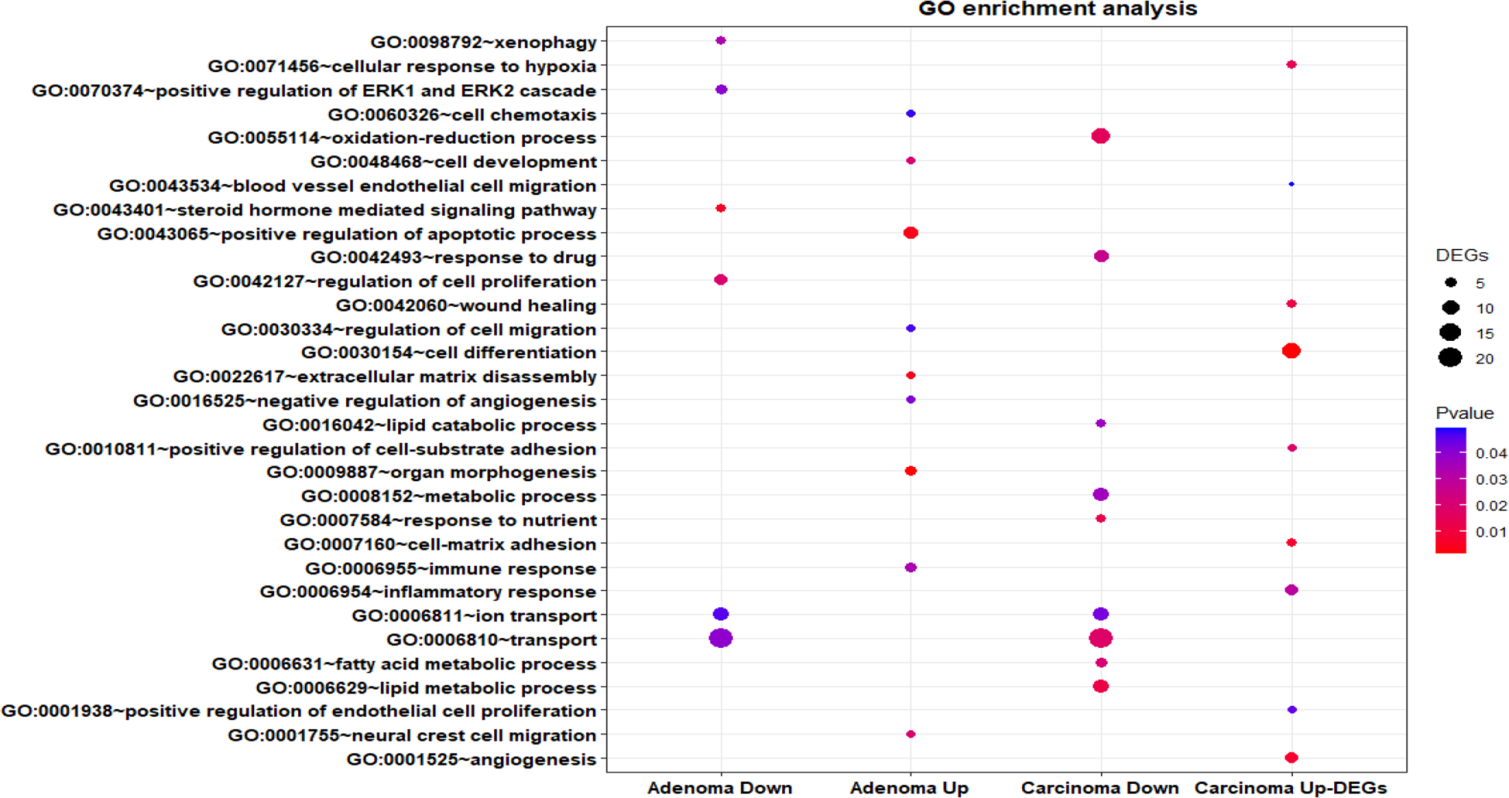
GO enrichment analysis of DEGs in the different stages of CRC. Dot plots showing GO terms in early-stage of CRC adenoma and late-stage carcinoma for upregulated and downregulated DEGs. GO, Gene Ontology; the size of circle shows the number of genes while the color shows *p*-value

GO analysis performed using DAVID showed that in the early stage of CRC (adenoma), the upregulated DEGs were mainly enriched in organ morphogenesis, positive regulation of apoptotic process, regulation of cell migration, and immune response, whereas downregulated DEGs referred to xenophagy, transport, and regulation of cell proliferation. In the carcinoma stage, the upregulated DEGs were significantly enriched in cell-matrix adhesion, cell differentiation, positive regulation of cell-substrate adhesion, and inflammatory response, while downregulated DEGs referred to transport, lipid metabolic process, oxidation-reduction process, and response to drug. These pathways in CS stages may vigorously induce the progression and metastasis of CRC (Fig. 3).

**Fig. 4:**
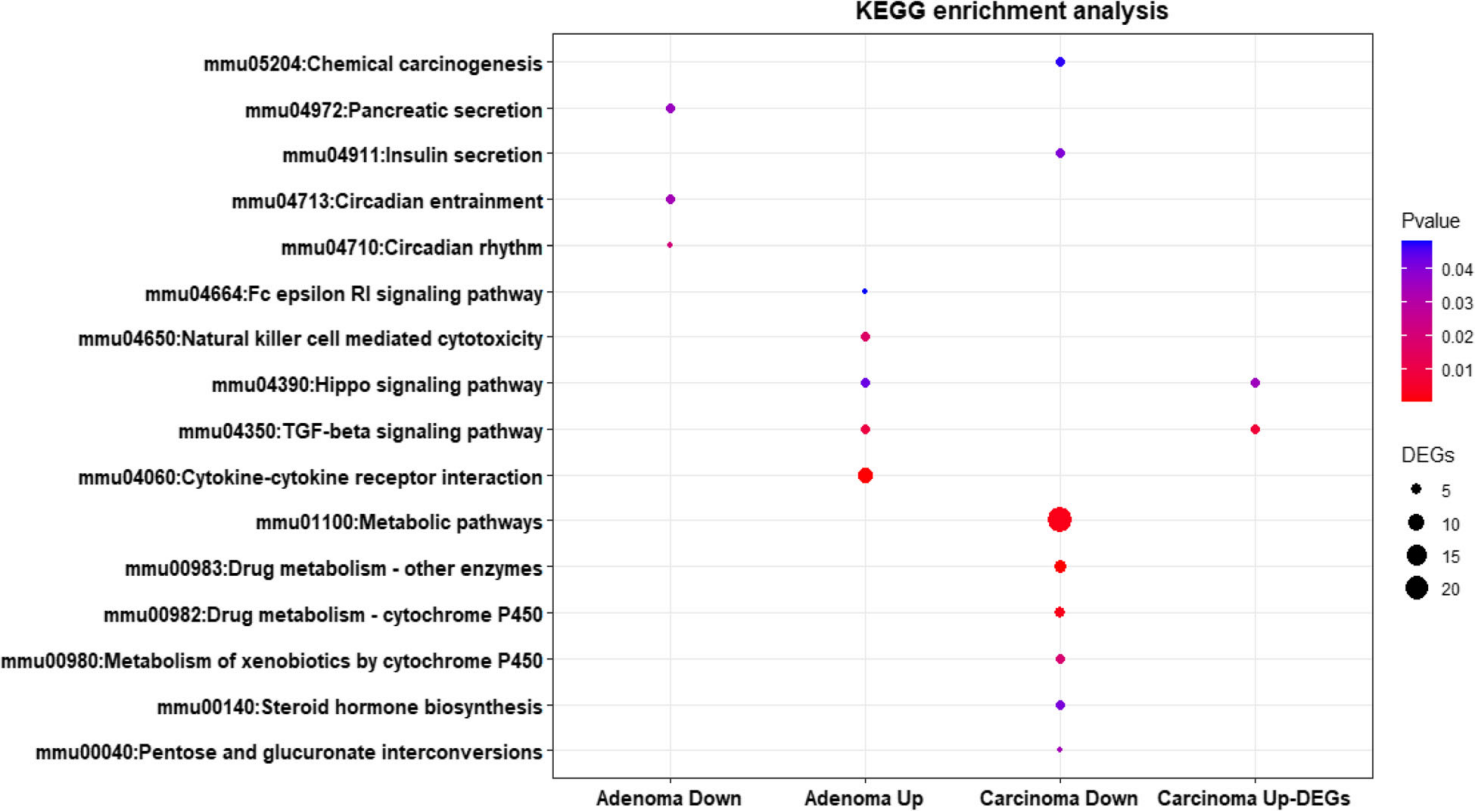
KEGG pathways enrichment analysis of DEGs in both stages of CRC. Dot plots showing KEGG pathways in early-stage of CRC adenoma and late-stage carcinoma. The size of circle shows the number of genes while the color shows *p*-value [38–40]

Figure 4 presents the significantly enriched KEGG [38–40] pathways of the upregulated and downregulated DEGs. For AS, the upregulated DEGs were significantly enriched in cytokine-cytokine receptor interaction, while the downregulated ones referred to circadian rhythm. In CS, the upregulated DEGs were significantly enriched in transforming growth factor-beta (TGF-beta) signaling pathway, while the downregulated ones referred to metabolic pathways and drug metabolism. These enriched terms and pathways may provide a new insight toward further research directions about the role of DEGs in colon cancer progression (Additional file 5).

### Mkp-1 modulates the key molecular processes typically related to tumorigenesis and cancer progression

**Fig. 5:**
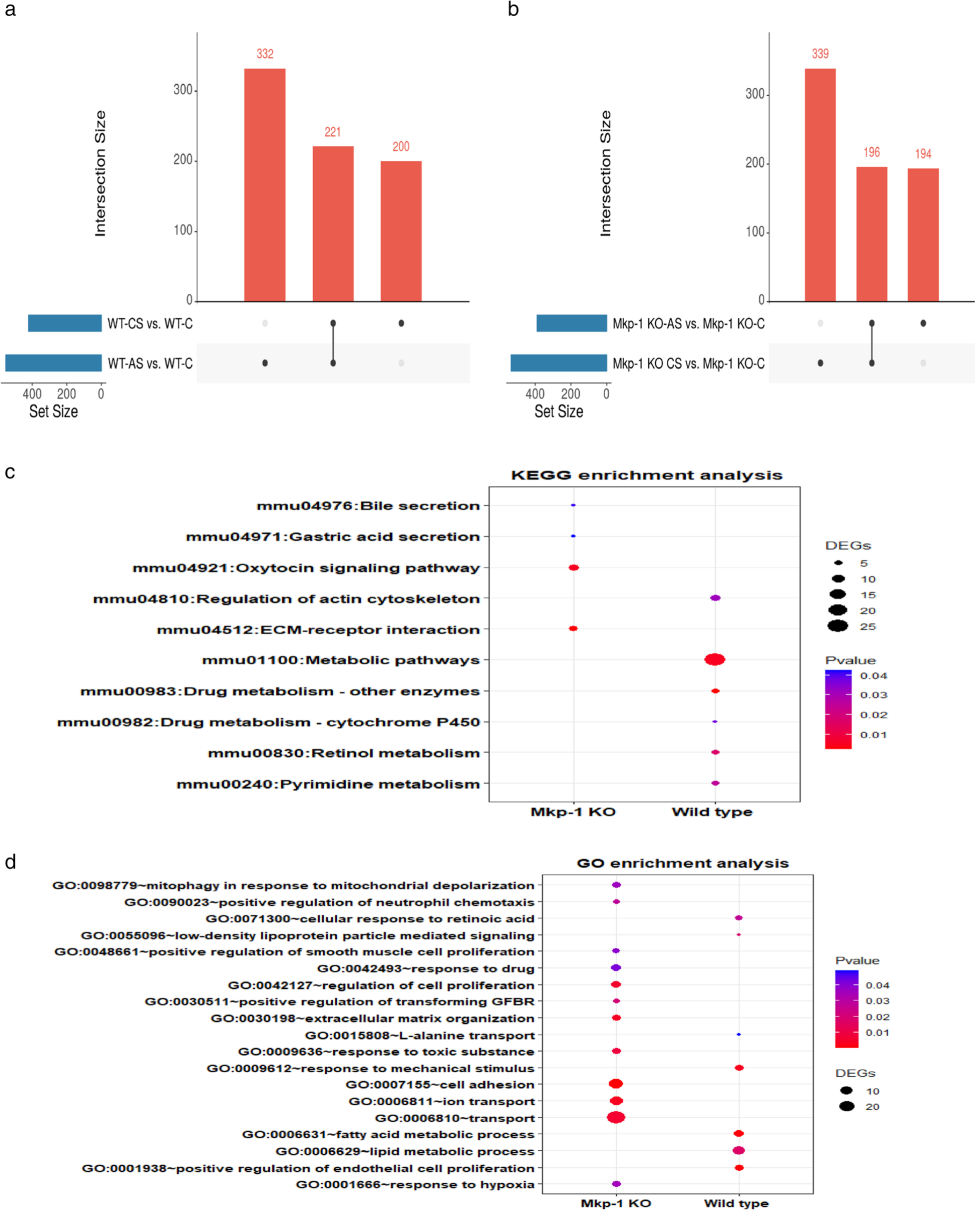
Mkp-1 knockdown alters gene profiling and targets in CRC. **a** UpSet plot showing overlapped DEGs in WT mice. **b** UpSet plot showing overlapped DEGs in KO mice. Each horizontal bar on the left represents the total number of DEGs identified in each set. Each vertical bar shows the number of genes that have been detected in each of the intersection sets, indicated by the connected points in the lower part of the plot. **c** Dot charts showing KEGG pathways comparison between KO and WT mice. **d** Dot plots showing GO terms in KO and WT mice. The size of circle shows the number of genes while the color shows *p*-value [38–40]

The DEGs identified by overlapping the DEGs specific for *Mkp-1*^*−/−*^in both stages in KO mice included 221 genes, while there are 196 genes for wild-type overlapping in both stages as we previously described [26] (Fig. 5a and b).

The GO terms and KEGG pathways analyses showed that there is a significant difference between wild-type and KO mice, in particular, the molecular signaling in KO mice was highly significant in cell adhesion, extracellular matrix organization, and regulation of cell proliferation. These results suggest that the *Mkp-1* gene could modulate the regulation of key molecular processes typically related to tumorigenesis and cancer progression (Fig. 5c and d, Additional file 6) [38–40].

### Nine hub genes regulated by Mkp-1

**Fig. 6:**
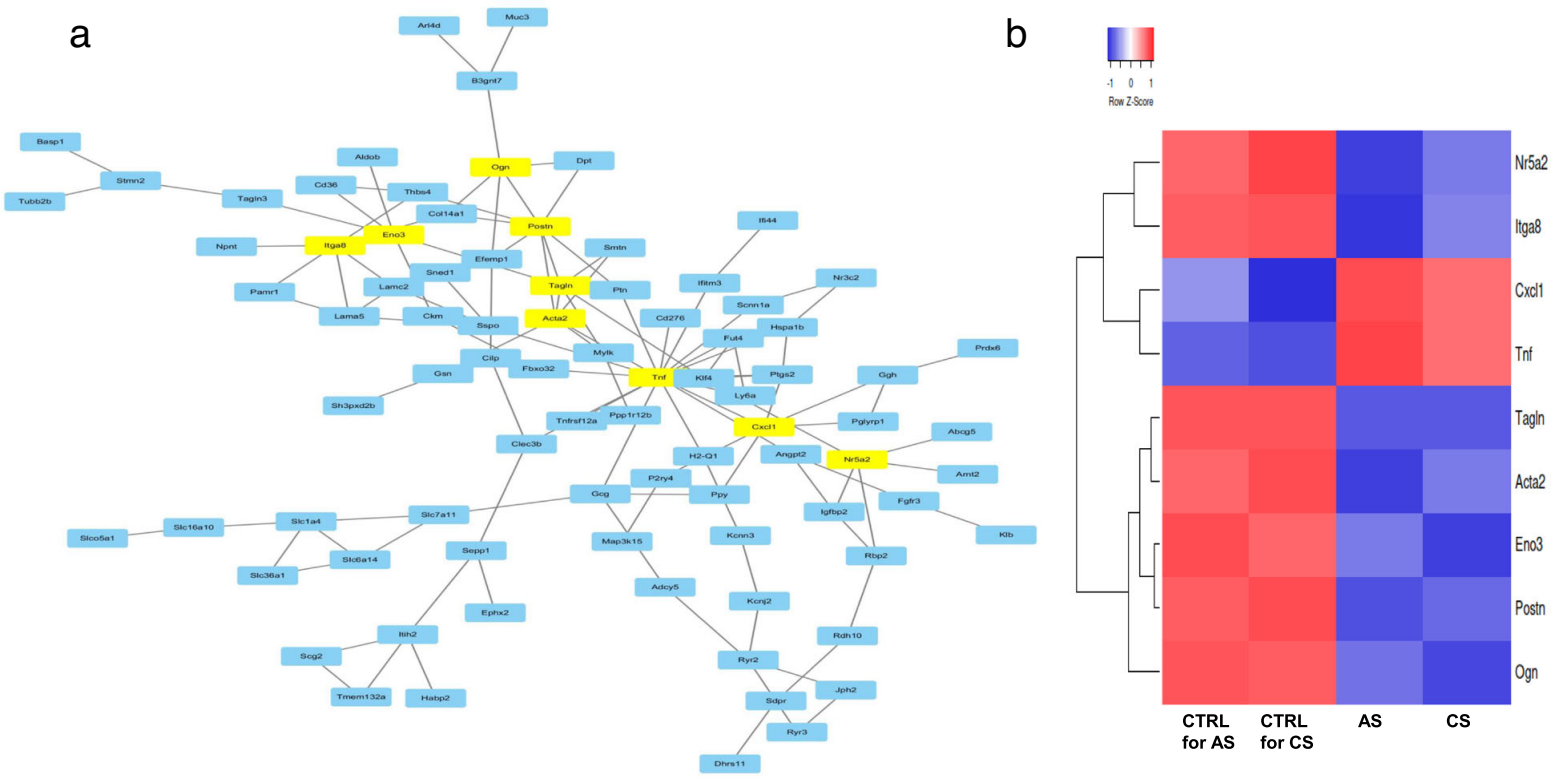
**a** PPI network of common DEGs using STRING. The protein–protein interaction (PPI) network constructed using the differentially expressed genes common to both adenoma KO and carcinoma KO. This network contained 194 nodes and 132 interaction pairs Tnf, Postn, Itga8, Acta2, Tagln, Cxcl1, Ogn, Eno3, and Nr5a2 had high degrees. **b** Heatmap for hub genes showing that most of them are down-regulated in KO mice

To further investigate the molecular mechanism of Mkp-1-promoted tumor growth and interactive relationships among all DEGs, we mapped the 196 DEGs to the STRING database, and validated interactions with a combined score of > 0.4 (high confidence) were selected to construct a PPI network. The PPI network consisted of 194 nodes and 132 interactions (Fig. 6a). In the PPI network, nine proteins, including tumor necrosis factor B (Tnf), periostin (Postn), integrin alpha-8 (Itga8), actin, aortic smooth muscle (Acta2), transgelin (Tagln), growth-regulated alpha protein (Cxcl1), mimecan (Ogn), beta-enolase (Eno3), and nuclear receptor subfamily 5 group A member 2 (Nr5a2) were strongly connected to other proteins (degree centrality> 5), indicating that they were hub genes (Fig. 6b). These hub genes might play crucial roles in the progression of CRC. The hub genes and their corresponding degrees are shown in Table 1. The GO functions and KEGG [38–40] pathways for hub genes are shown in Table 2.

**Table 1:** The top hub genes screened by Cytoscape web tool

Name	Degree	Betweenness Centrality	Closeness Centrality
**Tnf**	17	0.67993243	0.36681223
**Postn**	8	0.17053133	0.28093645
**Itga8**	6	0.04384682	0.21989529
**Acta2**	6	0.19197903	0.32061069
**Tagln**	6	0.1451616	0.28668942
**Cxcl1**	6	0.1185472	0.28474576
**Ogn**	5	0.07264773	0.23333333
**Eno3**	5	0.13239944	0.23728814
**Nr5a2**	5	0.08694301	0.23076923

**Table 2:** GO terms and KEGG pathways for hub genes screened by string web tool

Term	Term description	FDR
**GO:0009888**	tissue development	0.0014
**GO:0032722**	positive regulation of chemokine production	0.0016
**GO:0009653**	anatomical structure morphogenesis	0.0027
**GO:0030154**	cell differentiation	0.0027
**GO:0048856**	anatomical structure development	0.0027
**GO:2000343**	positive regulation of chemokine (C-X-C motif) ligand 2 production	0.0027
**GO:0050715**	positive regulation of cytokine secretion	0.003
**GO:0009887**	animal organ morphogenesis	0.0035
**GO:0009966**	regulation of signal transduction	0.0048
**GO:2000147**	positive regulation of cell motility	0.0055
**GO:0030198**	extracellular matrix organization	0.0056
**KEGG pathway**		
**mmu05134**	Legionellosis	0.0225
**mmu04657**	IL-17 signaling pathway	0.0228
**mmu04668**	TNF signaling pathway	0.0228
**mmu04621**	NOD-like receptor signaling pathway	0.0292
**mmu05205**	Proteoglycans in cancer	0.0364

### Nine hub genes that may be important in CRC prognosis

**Fig. 7:**
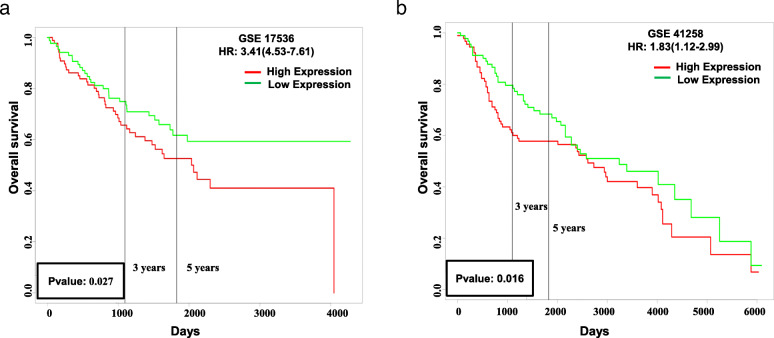
Prognostic values of 9 candidate genes in different CRC cohorts. **a** Cohort GSE 17536. **b** Cohort GSE 41258

Using the PROGgeneV2 database, it was shown that higher mRNA expression of 9 hub genes as a signature in 2 different cohorts, GSE17536 (HR: 3.41 [1.53–7.61]) and GSE41258 (HR: 1.83[1.12–2.99]), were related to poor survival in colon cancer patients (Fig. 7). Kaplan–Meier analysis of the overall and disease-free survival of colorectal cancer patients was based on the PrognoScan database (http://www.prognoscan.org/) using the publicly available Gene Expression Omnibus (http://www.ncbi.nlm.nih.gov/geo) with the accession number GSE 17536, GSE17537, and GSE12945, showing that these genes might be used in CRC prognosis (Additional file 2 (sFig. 2) and Additional file 7).

## Discussion

CRC can arise through the progression of adenoma, which is a consequence of genetic and epigenetic events in epithelial cells. In addition, the major causes of CRC development are Wnt/β-catenin activation and malignant transformation of inflammatory bowel disease [41–43], leading to increased proliferation and decreased apoptosis in the intestinal tract. Moreover, several recent studies have revealed that Mkp-1, a major negative regulator of the immune response, is overexpressed in CRC and associated with poor prognosis [15, 19]. However, the available data on the pathological role of Mkp-1 in CRC tumorigenesis are not clear and the molecular changes induced by MKp-1 in CRC have not been fully identified. Therefore, the objective of this study was to highlight the molecular signaling differences between CRC adenoma stage (AS) and carcinoma stage (CS), in addition to exploring the roles of Mkp-1 in CRC development by bioinformatics analyses using RNA-seq of WT and KO mice.

To better understand the function of DEGs in adenoma, GO analysis and KEGG pathway were performed. Upregulated genes mainly involved in biological processes consisting of organ morphogenesis, extracellular matrix disassembly, and cell development, which are essential for the initiation of tumors. Downregulated genes are also accumulated in biological processes that serve as barriers to cancer. For example, steroid hormone-mediated signaling pathways such as estrogens and their receptors may in fact exert an anti-tumor effect through selective activation of pro-apoptotic signaling, inhibition of inflammatory signals, and modulation of the tumor microenvironment [44]. In addition to autophagy, xenophagy is responsible for eliminating intestinal microbiota [45] that play a key role in CRC initiation and development; therefore, xenophagy inhibits colitis and CRC initiation [46]. Moreover, disruption of circadian rhythm is involved in increasing cancer risk, chemoresistance, and progression in CRC [47, 48]. Lastly, decreased expression of negative regulators such as PER2 and PER3 is associated with poorer differentiation, increased aggressiveness, and worse prognosis [49].

Regarding the function of DEGs in carcinoma, Upregulated genes were mainly involved in biological processes, including angiogenesis, cell migration, wound healing, cell differentiation, and inflammatory responses, which are essential for the maintenance of tumor development and metastasis [50, 51]. Downregulated genes were also found to participate in lipid metabolism, oxidation-reduction process, metabolic signaling, and drug metabolism, alteration of which is acquired by cancer to induce metabolic reprogramming and increase CRC progression [26]. Therefore, we may use the DEGs involved in the carcinoma stage found in this study to develop a novel strategy for CRC prognosis and prevent metastasis.

To address the hypothesis that Mkp-1 has critical roles in some cellular pathways required for tumor development, progression, and metastasis, we used a double strategy that combined RNA-seq and knockout technology. This strategy provided a differential expression profile of genes functionally related to Mkp-1. Here, we report that the disturbance of Mkp-1 expression in CRC induces changes in the expression levels of genes involved in tumor progression and metastasis, which may be a result of modulation of JNK, ERK and p38 activity [52]. In particular, Mkp-1 modulated key molecular processes typically activated in cancer, such as the regulation of extracellular organization, proliferation, cell adhesion, transforming growth factor-beta (TGF-β) signaling, and response to drugs. It was obvious that these pathways are closely associated with cancer development and metastasis (Fig. 5). Additionally, these results coincide with most recent results that indicate that Mkp-1 promotes angiogenesis and metastasis in lung cancer [52] and provide useful information for further research on the detailed molecular mechanisms of Mkp-1 in CRC development.

Networks identified hub nodes and interactions, which may aid in elucidating the underlying molecular mechanisms. The constructed PPI network based on STRING included 194 nodes and 132 edges. Nine DEGs were identified with degree> 5. These genes regulated by Mkp-1 were regarded as hub genes, and may play important roles in tumor development (Table 1). The results of this study revealed that the molecular processes regulated by these candidate genes are enriched in tissue development, positive regulation of chemokine production, cell differentiation, positive regulation of chemokines, positive regulation of cytokine secretion, organ morphogenesis, regulation of signal transduction, positive regulation of cell motility, and extracellular matrix organization. Moreover, the pathways identified in KEGG were connected to legionellosis, IL-17 signaling pathway, TNF signaling pathway, and proteoglycans in cancer (Table 1). These results are in agreement with previously published articles that showed that Mkp-1 involved in the regulation of the immune response, cell proliferation, and cell differentiation through its role in MAPK signaling pathways [12, 28].

We also found that some of these genes have been used as diagnostic biomarkers for CRC and other types of tumors; in particular, overexpression of Tagln is associated with the progression of colon cancer and may serve as a new biomarker for predicting the progression and prognosis of CRC [26, 53]. A previous study has shown that Postn expression in cancer cells promotes metastasis of CRC, by activating the PI3 kinase (PI3K)/protein kinase B (Akt) signaling pathway, which supported our results that showed that Mkp-1 may influence tumor progression and metastasis [54, 55] through regulation of Postn expression; Postn could also be used as a prognostic and predictive factor for CRC [54]. Mkp-1 induces CRC metastasis by regulating the expression of the Itga8 gene, a member of the integrin family pattern recognition receptors that participate in many cellular pathways, including adhesion and metastatic spread of tumor cells [56]. In addition, Itga8 expression may be a potential diagnostic marker of colon cancer [57]. Recent studies have shown that there is a correlation between early brain metastasis of lung cancer and the Acta2 gene mutation, and Acta2 might be a promising diagnostic and prognostic target for lung [58] and colon cancers [59]. More recent studies have shown that Nr5a2, the last one of the candidate genes in our study, controls the development of some types of cancer by promoting cancer cell proliferation by regulating cell cycle. In particular, Nr5a2 knockdown in colon cancer cell lines leads to an impairment in cell proliferation [60, 61].

These results indicate that hub genes are associated with cancer development; therefore, we performed survival analyses for these genes to explore their prognostic value in the survival of patients with colon cancer in different cohort studies. We identified a higher expression of candidate genes leads to poor survival in CRC patients. Overall, these findings suggest that hub genes may play a critical role in cancer development and may be a useful marker for predicting the survival of cancer patients and may be significant in determining treatment outcomes.

## Conclusion

Our study revealed that there are differences in molecular pathways between different stages of CRC. Therefore, our findings regarding differences in DEG between adenomas and carcinomas and their molecular targets involved in this study could help in early determination of the adenoma stage and may decrease or prevent CRC progression. Additionally, Mkp-1 plays a crucial role in CRC tumorigenesis by remodeling pathways related to cell proliferation, metastasis, and immune responses. Moreover, by considering their limitations, we suggest that hub genes identified in this work may be used in the prognosis of CRC. Functional investigations of the mechanisms related to these genes are necessary.

## Supplementary Information

**Additional file 1.** ARRIVE Guidelines checklist.

**Additional file 2:** **sFig 1.** Volcano plots and **sFig 2.** Survival curves, Prognostic values of some candidate genes in CRC different cohort.

**Additional file 3.** The list of DEGs for adenoma stage in wild type and Mkp-1 knockout mice.

**Additional file 4.** The list of DEGs for carcinoma stage in wild type and Mkp-1 knockout mice.

**Additional file 5.** The enrichment analysis for common DEGs between wild type and Mkp-1 knockout mice.

**Additional file 6.** The list of GO terms and KEGG pathways wild type and Mkp-1 knockout mice.

**Additional file 7.** The survival analysis of the overall and disease-free survival of colorectal cancer patients based on the PrognoScan database.

## Data Availability

The RNA-seq data of raw and processed files have been deposited in NCBI Gene Expression Omnibus, accession number GSE164960 (https://www.ncbi.nlm.nih.gov/geo/query/acc.cgi?acc=GSE164960).

## Abbreviations

CRC: Colorectal cancer
Mkp-1: Mitogen-activated protein kinase phosphatase-1
RNA seq: RNA sequencing
KO: Knockout
AOM: Azoxymethane
DSS: Dextran sodium sulfate
DEGs: Differentially expressed genes
GO: Gene Ontology
KEGG: Kyoto Enrichment of Genes and Genomes
PPI: Protein protein interaction
TGF-beta: Transforming growth factor beta
ECM: Extracellular matrix
WT: Wild type
AS: Adenoma stage
CS: Carcinoma stage
H&E: Hematoxylin and eosin
C: Control
FPKM: Kilobase of exon model per Million mapped reads
DAVID: The NIH Database for Annotation, Visualization and /??/Integrated Discovery
Tnf: Tumor necrosis factor B
Postn: Periostin
Itga8: Integrin alpha-8
Acta2: Actin, aortic smooth muscle
Tagln: Transgelin
Cxcl1: Growth-regulated alpha protein
Ogn: Mimecan
Eno3: Beta-enolase
Nr5a2: Nuclear receptor subfamily 5 group A member 2
