# Supplementary material for: Transcriptome analysis of potential candidate genes and molecular pathways in colitis-associated colorectal cancer of Mkp-1-deficient mice
Source: BMC Cancer. 2021 May 25;21:607. doi: 10.1186/s12885-021-08200-0 (PMC8152130; doi:10.1186/s12885-021-08200-0)
Supplement: Supplementary file 2 — Additional file 2: sFig 1. Volcano plots and sFig 2. Survival curves, Prognostic values of some candidate genes in CRC different cohort. [file 12885_2021_8200_MOESM2_ESM.docx]

**
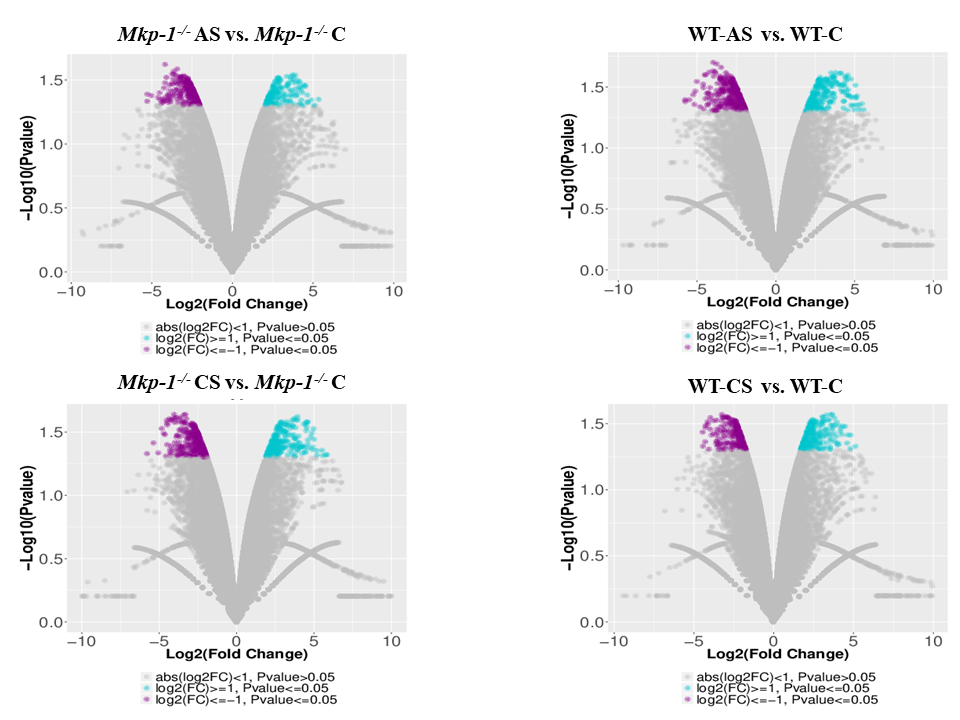
 sFig 1: Volcano plots**

**
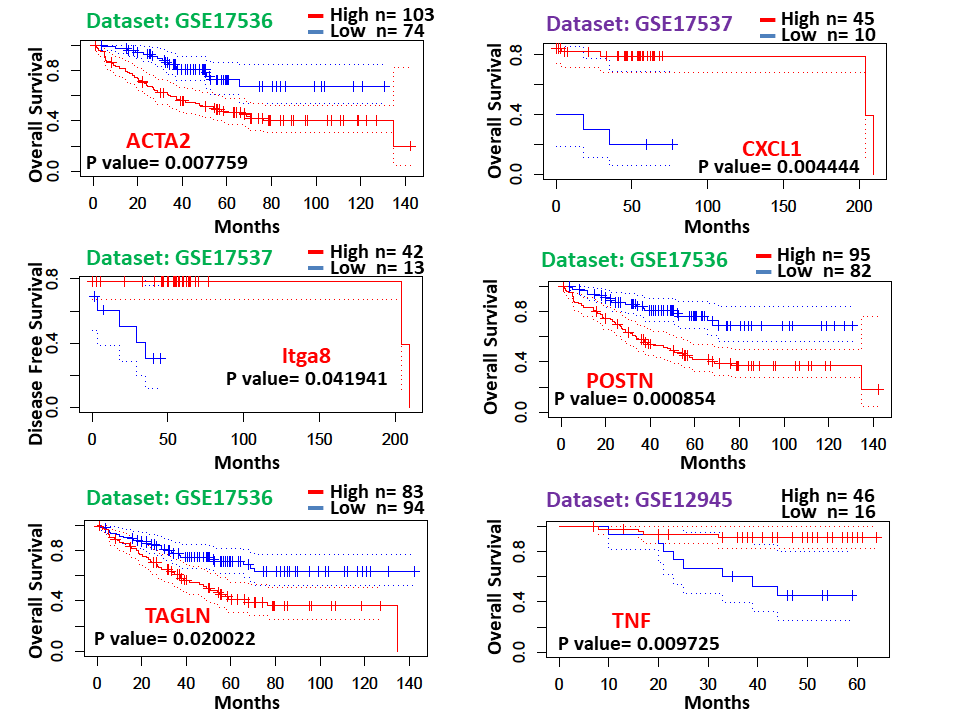
 sFig 2: survival curves, Prognostic values of some candidate genes in CRC different cohort.**
